# An innovative endovascular approach for acute ischemic stroke attributable to ICAS-LVO: the RESCUE (Retrievable stent-Endovascular tirofiban-paSs-ExtraCtion-microgUide-rEscue angioplasty) technique

**DOI:** 10.3389/fneur.2026.1886448

**Published:** 2026-08-28

**Authors:** Yingchun Wu, Hanzhang Wang, Yuejiang Gui, Rui Sun, Guanqing Feng, Wenzhao Li, Yanan Zheng, Wen Liu, Zhipeng Hua

**Affiliations:** Department of Neurology, ORDOS Central Hospital, Ordos, China

**Keywords:** intracranial atherosclerotic stenosis, large vessel occlusion, mechanical thrombectomy, rescue technique, stent retriever, tirofiban

## Abstract

**Background:**

Conventional mechanical thrombectomy (MT) for acute ischemic stroke (AIS) due to intracranial atherosclerosis-related large vessel occlusion (ICAS-LVO) is associated with low first-pass reperfusion (FPR), distal embolization, and recurrent thrombosis. We designed a novel stepwise endovascular strategy, the RESCUE (Retrievable stent-Endovascular tirofiban-paSs-ExtraCtion-microgUide-rEscue angioplasty) technique, and compared its safety and efficacy with that of the standard Solumbra technique.

**Methods:**

In this prospective, randomized, single-center trial, 85 patients presenting with AIS due to ICAS-LVO were enrolled consecutively. Eligible participants underwent randomization to either the RESCUE group (43 patients) or Solumbra group (42 patients). The primary endpoint was FPR, defined as achieving modified Thrombolysis in Cerebral Infarction (mTICI) grade 2c-3. Secondary endpoints comprised: favorable 90-day functional status [modified Rankin Scale (mRS) score ≤2 for anterior strokes or ≤3 for posterior strokes]; procedural duration from groin puncture to first recanalization; number of retrieval attempts; distal embolization; early reocclusion; symptomatic intracranial hemorrhage (sICH); and death within 90 days.

**Results:**

A significant difference in FPR rates was observed between the two groups, favoring the RESCUE group [86.0% (37/43) vs. 61.9% (26/42); *p* = 0.011]. The RESCUE group had a shorter median procedural duration [40.0 min (interquartile range (IQR) 30.0–50.0 min) vs. 60.0 min (IQR 43.0–77.0 min); *p* = 0.010]. Successful reperfusion, defined as mTICI grade ≥2b, occurred in 93.0% of patients in the RESCUE group and 90.5% of those in the Solumbra group (*p* = 0.373). At the 90-day follow-up, a favorable functional outcome was noted in 60.5% (26/43) of RESCUE group patients and 57.1% (24/42) of Solumbra group patients (*p* = 0.483). No significant between-group differences emerged in the incidence of sICH (2.3% vs. 4.8%, *p* = 1.000) or 90-day mortality (0% vs. 4.8%, *p* = 0.237).

**Conclusion:**

The RESCUE technique is a safe and effective lesion-specific endovascular strategy for ICAS-LVO, providing superior FPR and shorter procedural time compared with the conventional Solumbra technique, without increasing safety concerns. Larger-scale multicenter investigations are needed to confirm these results.

## Introduction

Among the various subtypes of acute ischemic stroke (AIS), intracranial atherosclerosis-related large vessel occlusion (ICAS-LVO) stands out as particularly challenging and accounts for a substantial proportion of cases, especially in Asian, Hispanic, and African American populations (1, 2). Accumulating evidence indicates that ICAS-LVO is associated with higher procedural failure rates, increased risk of reocclusion, and poorer long-term functional outcomes compared with embolic LVO (3, 4). The epidemiological impact is particularly pronounced in East Asian nations, where ICAS-LVO accounts for as much as 40–50% of all intracranial large vessel blockages, thereby presenting a major obstacle for endovascular therapy (1, 5).

Unlike cardiogenic embolic stroke, the pathological hallmark of ICAS-LVO is *in situ* thrombotic occlusion superimposed upon a pre-existing, high-grade atherosclerotic stenosis (3, 6). This unique pathophysiology creates a “resistant” lesion that often fails to respond to conventional mechanical thrombectomy (MT) techniques. The underlying stenotic plaque not only predisposes patients to recurrent thrombosis but also hinders complete clot retrieval and sustained vessel patency (7).

When only stent retrievers or aspiration catheters are employed, conventional MT often fails to establish sustained vessel patency in patients with ICAS-LVO. According to a systematic review, even when initial reperfusion is successful [modified Thrombolysis in Cerebral Infarction (mTICI) grade ≥2b], nearly half of ICAS-LVO patients develop early symptomatic reocclusion within 24 h, although this event rarely occurs in strokes of cardioembolic origin (4, 8). This high reocclusion rate is primarily attributable to the inability of standard thrombectomy to address the underlying atherosclerotic stenosis, leading to persistent flow-limiting residual stenosis and a prothrombotic milieu (3, 9).

To address these shortcomings, multiple “rescue” approaches have been incorporated into clinical practice, such as intra-arterial delivery of glycoprotein IIb/IIIa inhibitors (e.g., tirofiban), balloon angioplasty, and intracranial stenting (9). Tirofiban is a rapidly acting, reversible non-peptide inhibitor of the platelet glycoprotein IIb/IIIa receptor. Local administration of this agent has demonstrated potential in diminishing residual thrombus burden and avoiding reocclusion (10, 11). However, the optimal route (intravenous vs. intra-arterial), timing, and dosing of tirofiban remain debated. The recent RESCUE BT randomized trial showed that routine intravenous administration of tirofiban before MT did not enhance functional outcomes in unselected AIS patients; however, subgroup analyses indicated a possible benefit for those with ICAS-LVO (12, 13). Conversely, several single-center studies have reported encouraging results with local intra-arterial tirofiban infusion after failed thrombectomy, achieving higher recanalization rates without significantly increasing symptomatic intracranial hemorrhage (sICH) (11, 14). Balloon angioplasty and stenting can effectively address the underlying stenosis; however, these interventions carry elevated risks of vessel dissection, perforation, and sICH, especially when carried out on an emergency basis (15, 16). The recent multicenter ANGEL-REBOOT trial specifically addressed endovascular bailout strategies for ICAS-LVO and found that while rescue angioplasty/stenting achieved acceptable recanalization rates, it also carried a notable risk of procedural complications (10).

Despite these advances, current rescue therapies are often applied empirically without a standardized, stepwise protocol. This lack of procedural uniformity leads to inconsistent outcomes, variable complication rates, and difficulty in comparing results across studies (9, 10). Therefore, there is an urgent need for a structured, lesion-specific endovascular strategy that integrates pharmacological and mechanical approaches in a logical sequence to maximize efficacy while minimizing risks.

To fill this clinical void, our institution independently devised a new standardized endovascular protocol for patients with ICAS-LVO, named the RESCUE (Retrievable stent-Endovascular tirofiban-paSs-ExtraCtion-microgUide-rEscue angioplasty) technique. This technique sequentially combines: (1) immediate flow restoration via stent retriever deployment; (2) focal intra-arterial tirofiban infusion to enhance local antiplatelet effects and soften atherothrombotic debris; (3) active aspiration extraction with distal stent retriever protection; (4) pre-wiring of the occluded segment for rapid subsequent intervention; and (5) bailout angioplasty/stenting if significant residual stenosis persists. The rationale is to provide a comprehensive yet stepwise “all-in-one” approach that addresses both the acute thrombus and the underlying plaque.

This prospective, randomized study aimed to systematically compare the safety and efficacy of the RESCUE technique versus the conventional Solumbra technique in AIS patients with ICAS-LVO, ultimately seeking to establish a novel standardized treatment paradigm for this complex stroke population.

## Materials and methods

### Study design

This prospective, randomized, single-center trial was approved by the Institutional Ethics Committee of Ordos Central Hospital. Consecutive patients with acute ischemic stroke (AIS) caused by intracranial atherosclerosis-related large vessel occlusion (ICAS-LVO) who were eligible for endovascular thrombectomy were prospectively enrolled from the Department of Neurology of Ordos Central Hospital between January 2023 and December 2025.

An internal data monitoring panel consisting of independent stroke neurologists was established to conduct regular data review meetings, acting as an informal Data Monitoring Committee (DMC). All clinical and procedural data were recorded via standardized electronic case report forms (CRFs), with mandatory dual independent cross-verification performed for all variables. We have also fully documented the protocol for centralized sealed-envelope randomization with strict allocation concealment.

**Figure 1:**
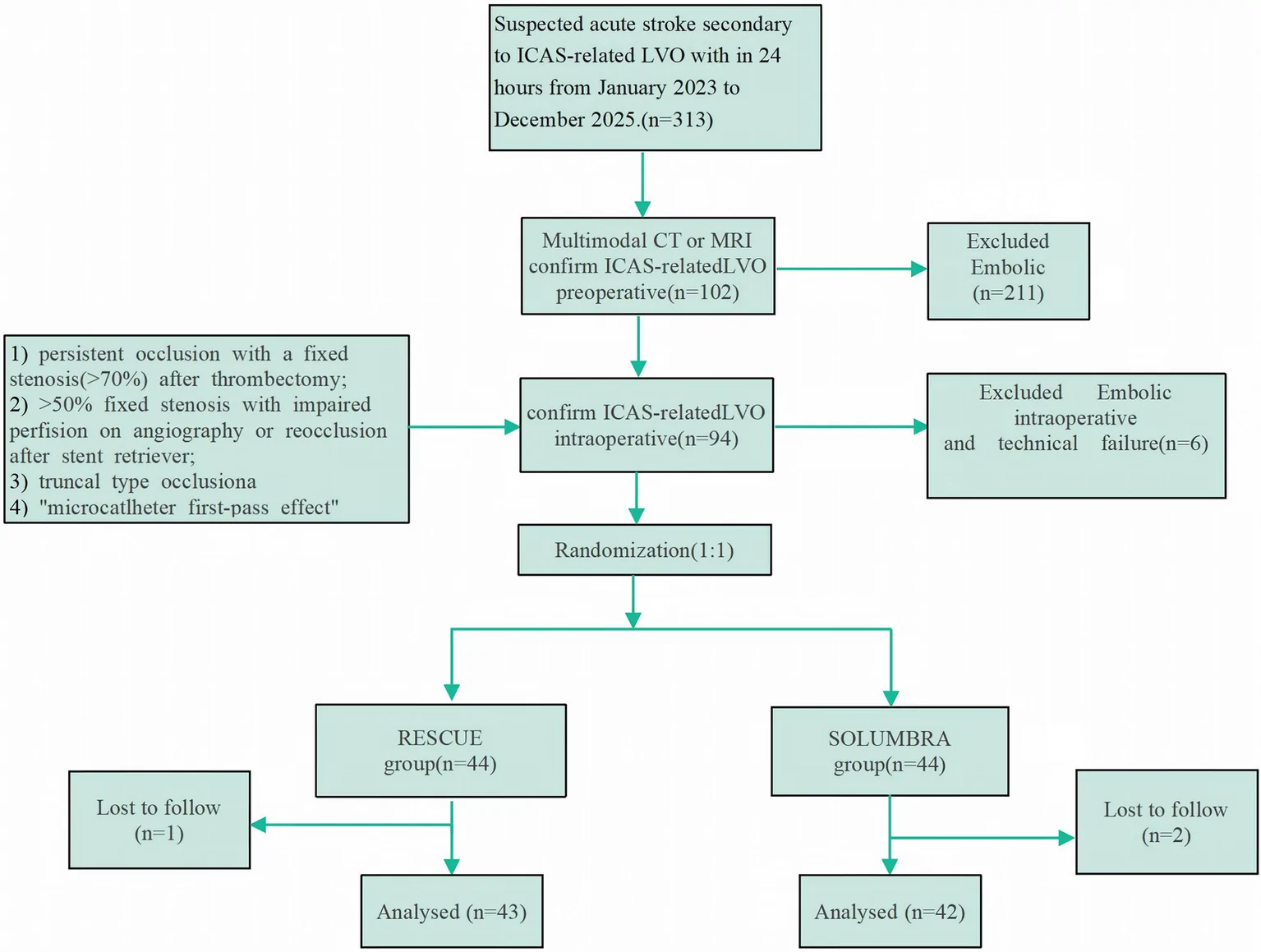
Study flowchart. A total of 313 patients with suspected ICAS-related LVO were screened. After multimodal imaging and intraoperative confirmation, 94 patients were identified as having ICAS-LVO. Four patients were excluded postoperatively, and the remaining 88 were randomized 1:1 to the RESCUE group (*n* = 44) or the Solumbra group (*n* = 44). After losses to follow-up, 43 patients in the RESCUE group and 42 in the Solumbra group were included in the final analysis.

Eligible patients were assigned to either the RESCUE group (*n* = 43) receiving the RESCUE technique or the control Solumbra group (*n* = 42) via simple coin-toss randomization. All study endpoints were evaluated by two experienced neurologists who remained blinded to group assignments. The trial flowchart is presented in Figure 1.

### Patient selection

The following criteria were required for inclusion: (1) age ≥18 years; (2) angiographic evidence (via magnetic resonance angiography, computed tomography angiography, or digital substraction angiography) of occlusion in the intracranial internal carotid artery, the M1 segment of the middle cerebral artery, the intracranial vertebral artery (V4), or the basilar artery (BA); (3) symptom onset to groin puncture interval less than 24 h; (4) pre-stroke modified Rankin Scale (mRS) score <3; and (5) baseline National Institutes of Health Stroke Scale (NIHSS) score of ≥6.

Exclusion criteria comprised intracranial hemorrhage detected by computed tomography or magnetic resonance imaging (MRI) during the 3 weeks prior to enrollment. All patients or their legally authorized representatives gave written informed consent.

### Evaluation of ICAS-related LVO

The diagnosis of ICAS-LVO followed previously established criteria (10, 17), which included the following: (1) presence of conventional atherosclerotic risk factors; (2) absence of atrial fibrillation; (3) occlusion of a major arterial trunk; (4) tapered morphology of the occlusion stump; (5) absence of the susceptibility vessel sign on MRI; (6) deformity of stent struts; (7) microcatheter first-pass effect; (8) visible “waist” upon stent deployment (stent-unsheathed effect); and (9) residual fixed stenosis exceeding 70% with persistent occlusion after the initial thrombectomy. ICAS-LVO is diagnosed if all four core criteria (criteria 1–4) are met, together with at least two of the auxiliary criteria (criteria 5–9).

### Imaging results and clinical evaluation

All imaging data were independently evaluated by two experienced neurologists who were unaware of the clinical information. The collected variables encompassed baseline demographic data, vascular risk factors, admission NIHSS score, use of intravenous alteplase (tPA), time from symptom onset to groin puncture, procedure time (defined as groin puncture to first recanalization, in minutes), thrombectomy technique, number of passes, use of angioplasty or stenting, type of stent, procedural complications, and symptomatic intracranial hemorrhage (sICH). Consistent with the European Cooperative Acute Stroke Study II (ECASS II) criteria (18), sICH was defined as any intracranial hemorrhage (including parenchymal hematoma, subarachnoid hemorrhage, or intraventricular hemorrhage) accompanied by a neurological deterioration of ≥4 points on the NIHSS. Successful revascularization was defined as achieving mTICI grade 2b-3. At 90 days, follow-up data were gathered by two independent physicians blinded to the treatment assignments. A favorable long-term prognosis was defined as a 90-day mRS score of ≤2 for anterior circulation strokes and ≤3 for posterior circulation strokes (19).

### Endovascular therapy and the Solumbra technique

All endovascular interventions were carried out under general anesthesia through a transfemoral route. A 6F long sheath was placed retrograde into the femoral artery, after which diagnostic digital subtraction angiography was performed. Once the guiding catheter was positioned in the internal carotid artery (ICA) or vertebral artery (VA), an aspiration catheter was advanced into the target vessel, and the Solumbra technique was subsequently executed (6). More precisely, 5 min after deployment of the stent retriever, the aspiration catheter was advanced to the thrombus–vessel interface, and manual suction was generated using a 50-mL locked syringe. Following this, both the aspiration catheter and the stent retriever were withdrawn together into the cervical guiding catheter, thus removing the thrombus.

### RESCUE technique

**Figure 2:**
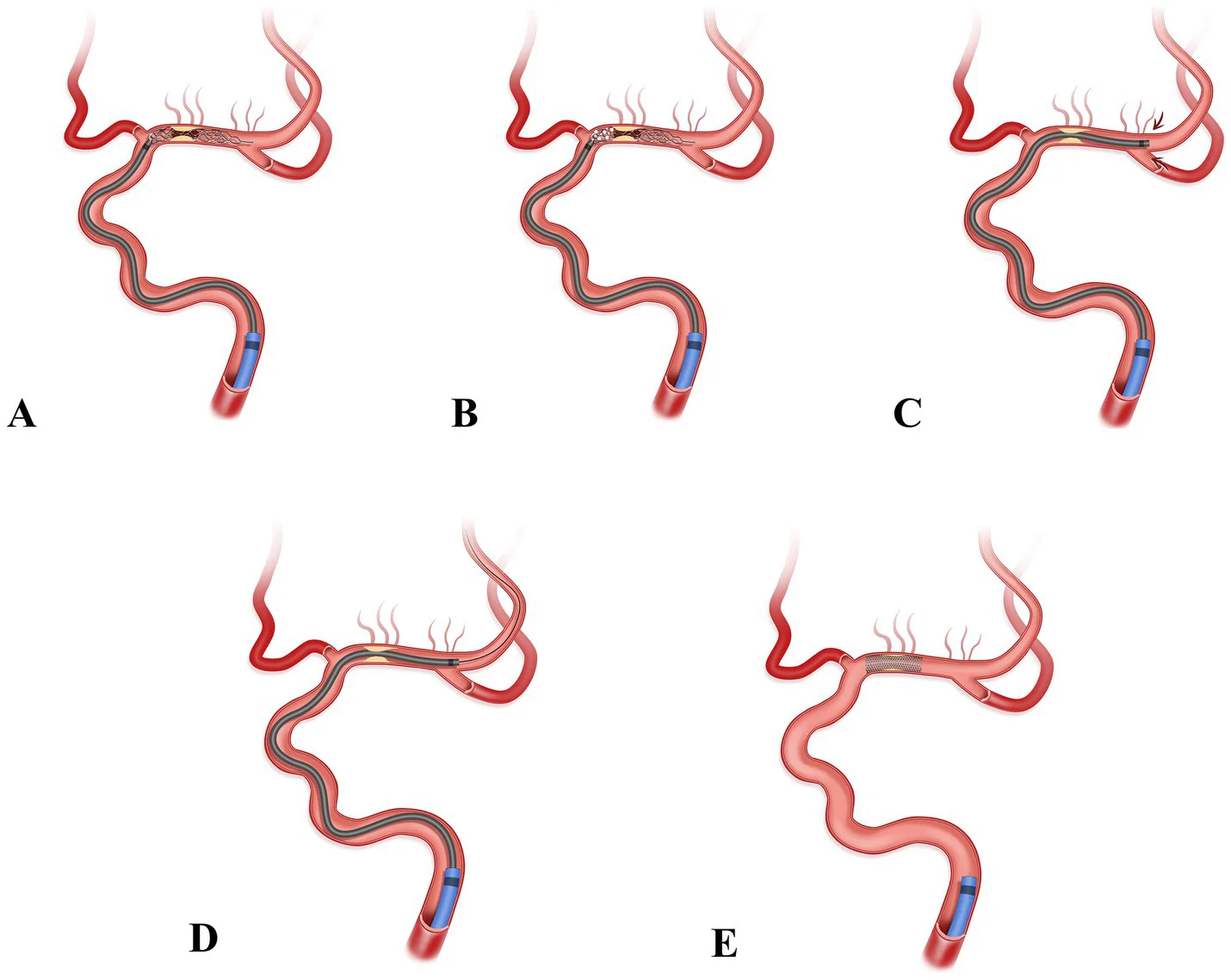
Schematic illustration of the RESCUE technique. **(A)** The stent retriever is deployed across the lesion to create a bypass channel. **(B)** Focal endovascular tirofiban is infused for 10 min to enhance regional antiplatelet effects and soften residual atherothrombotic debris. **(C)** The aspiration catheter is advanced through the thrombus with distal stent retriever protection. Once blood flow is maintained, >50 mL of blood is aspirated to ensure that the withdrawn blood contains no thrombus. **(D)** A 300-cm microguidewire is passed through the occlusion into the distal segment under the guidance of the aspiration catheter. **(E)** Angiography is performed after the aspiration catheter is withdrawn proximal to the occlusion, if balloon angioplasty or stenting is performed if necessary.

The RESCUE technique is first described in the present study. The procedure was performed sequentially as follows (Figure 2). A stent retriever was initially placed across the lesion to establish a bypass channel (Figure 2A). Next, focal endovascular tirofiban was infused for 10 min to enhance regional antiplatelet effects and soften residual atherothrombotic debris (Figure 2B). The aspiration catheter was subsequently advanced through the occluding thrombus, following the distal tip of the stent retriever (Figure 2C). Once stable blood flow was established, more than 50 mL of blood was aspirated to confirm that no thrombus remained in the withdrawn blood. Next, a 300-cm microguidewire was navigated past the occlusion into the distal vessel segment under guidance of the aspiration catheter (Figure 2D). After the aspiration catheter was retracted to a position proximal to the occlusion, angiography was performed (Figure 2E). Finally, when indicated, balloon angioplasty or stenting was carried out.

In patients receiving tirofiban, a local rescue dose of 0.5–2.0 mg was administered. For intra-arterial infusion, 0.5 mg (2 mL) of tirofiban was combined with 8 mL of normal saline, or 1 mg (4 mL) with 6 mL of normal saline, resulting in a 10 mL solution. The diluted tirofiban was manually infused at an approximate rate of 1 mL/min (6). Intravenous tirofiban was continued at a rate of 0·1 μg/kg per min for 24 h. Oral antiplatelet therapy.

and intravenous tirofiban overlapped for 4 h, and then intravenous tirofiban was discontinued. Dual antiplatelet therapy with aspirin (100 mg per day) and clopidogrel (75 mg per day) was given orally for at least 3 months if no intracranial haemorrhage was observed.

### Statistical analysis

The Kolmogorov–Smirnov test was used to evaluate the normality of continuous data. Variables following a normal distribution are reported as mean ± standard deviation (SD), with intergroup comparisons conducted using Student’s *t*-test. For non-normally distributed continuous variables, results are presented as median and interquartile range [M (IQR)], and groups were compared using the Wilcoxon rank-sum test. Categorical and ordinal variables are expressed as frequencies (percentages) and were compared between groups via the Chi-square test. A two-sided *p* value <0.05 was deemed statistically significant. The FPR was defined as the primary endpoint. Based on our preliminary clinical data and published peer-reviewed literature, we estimated that the rate of successful reperfusion was 70.0% in the conventional Solumbra group and 92.0% in the RESCUE group. We adopted the classic formula for comparing two independent sample rates for sample size calculation: $n=\frac{2\bar{p}(1-\bar{p}){\left(Z_{1-\alpha /2}+Z_{1-\beta }\right)}^{2}}{{\left(p_{1}-p_{2}\right)}^{2}}$ . With a two-sided significance level of *α* = 0.05 (*Z* = 1.96) and a statistical power of 80% (1 − *β* = 0.80, *Z* = 0.84), the calculated minimum sample size was 38 cases per group, totaling 76 cases. Considering a 10% potential follow-up dropout rate, the final required sample size was 84 cases. Our study enrolled 85 patients in total, which exceeded the theoretical sample size and achieved an actual statistical power of 81.2%. This verified that the current sample size was statistically sufficient and credible for detecting intergroup differences in primary and secondary outcomes. All detailed calculation parameters, formulas, and power verification results have been completely supplemented in the revised manuscript.

## Results

### Baseline characteristics

At our institution, 85 patients with AIS received treatment with thrombectomy [71 men, 14 women; median age 63.0 years (IQR 54.0–70.0)]. The median baseline NIHSS score was 15.0 (IQR 11.0–18.0). Table 1 summarizes the demographic and clinical characteristics of the study population. No significant intergroup differences were found in age, sex, vascular risk factors (including hypertension, diabetes mellitus, hyperlipidemia, atrial fibrillation, coronary artery disease, prior ischemic stroke, and smoking), admission NIHSS score, diffusion weighted imaging (DWI)–Alberta Stroke Program Early CT Score (ASPECTS), intravenous thrombolysis, or occlusion location. The two groups also showed similar values for time from symptom onset to groin puncture, use of rescue endovascular therapy (balloon angioplasty and/or stenting) for ICAS, and embolization rate. No procedure-related complications or embolization events occurred in the RESCUE group.

**Table 1:** Baseline demographic and clinical characteristics of the study population.

Variables	Total (*n* = 85)	RESCUE group (*n* = 43)	Solumbra group (*n* = 42)	*p*-value
Age, years, (IQR)	63.00 (54.00, 70.00)	62.00 (54.00, 68.00)	63.00 (54.00, 71.00)	0.589
Male, *n* (%)	71 (83.5)	39 (90.7)	32 (76.2)	0.131
Hypertension, *n* (%)	47 (55.3)	24 (55.8)	23 (54.8)	1.000
Diabetes mellitus, *n* (%)	20 (23.5)	12 (27.9)	8 (19.0)	0.480
Dyslipidemia, *n* (%)	2 (2.4)	1 (2.3)	1 (2.4)	1.000
Current smoker, *n* (%)	27 (31.8)	14 (32.6)	13 (31.0)	1.000
Atrial fibrillation, *n* (%)	8 (9.4)	3 (7.0)	5 (11.9)	0.483
Baseline NIHSS score, median (IQR)	15.00 (11.0, 18.0)	15.00 (10.0, 16.0)	15.00 (12.0, 19.0)	0.237
Occlusion location, *n* (%)				
Anterior circulation (MCA/ICA)	71 (83.5)	35 (81.4)	36 (85.7)	0.807
Posterior circulation (BA/VA)	14 (15.3)	8 (18.6)	6 (14.3)	0.807
ASPECTS, median (IQR)	7.00 (7.0, 8.0)	7.00 (7.0, 8.00)	7.00 (7.0, 8.0)	0.369
Onset to groin time, min, median (IQR)	430.00 (130.00, 710.00)	487.00 (140.00, 639.00)	412.00 (129.00, 710.00)	0.52
Puncture to first recanalization time, min, median (IQR)	52 (33.0, 77.0)	40.0 (30.00, 50.00)	60.0 (43.0, 77.0)	0.010*
Intravenous thrombolysis, *n* (%)	24 (28.2)	10 (23.3)	14 (33.3)	0.429

ASPECTS, Alberta Stroke Program Early CT Score; IQR, interquartile range; MCA, middle cerebral artery; ICA, internal carotid artery; BA, basilar artery; VA, vertebral artery.

^*^Indicates statistical significance (*p* < 0.05).

### Procedural and clinical outcomes

**Figure 3:**
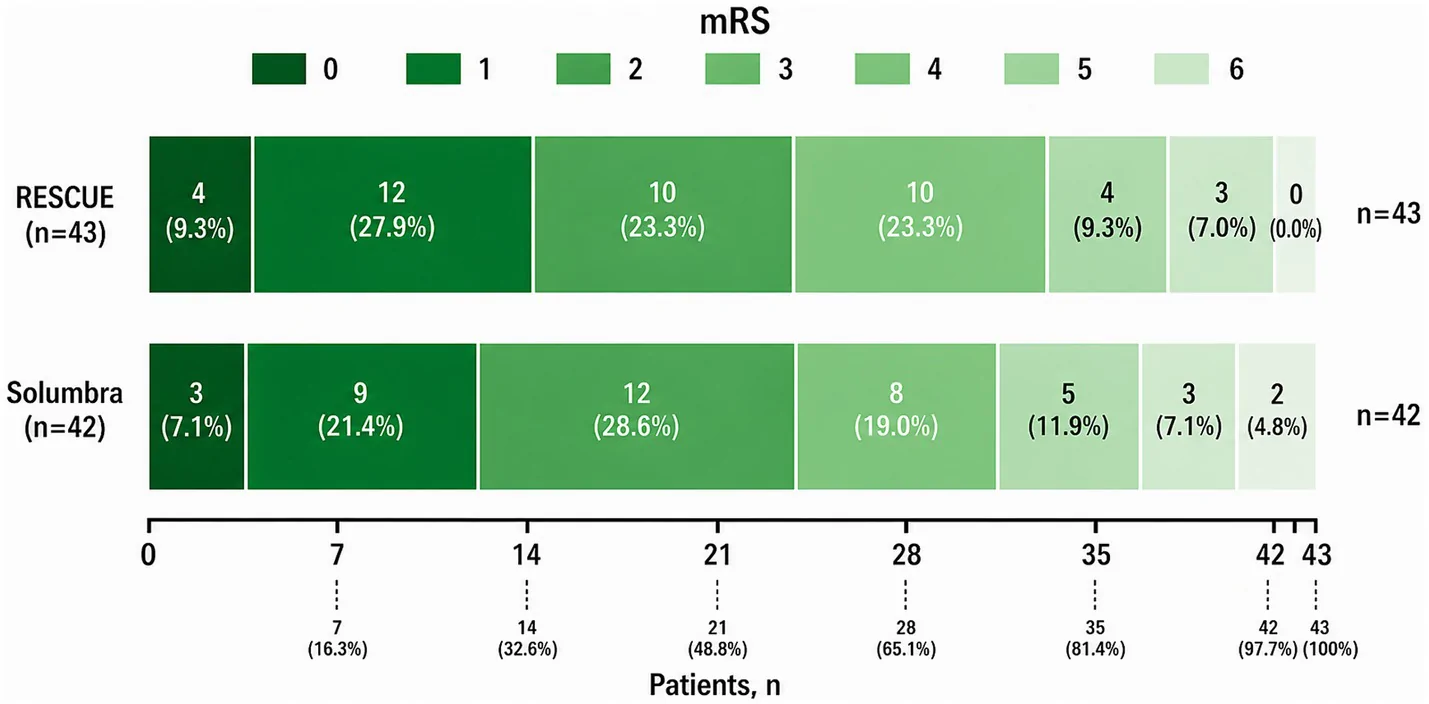
Distribution of scores on modified Rankin Scale at 90 days between two groups.

The median interval from femoral access to first recanalization was significantly shorter in the RESCUE group than in the Solumbra group [40.0 min (IQR 30.0–50.0 min) vs. 60.0 min (IQR 43.0–77.0 min), *p* = 0.01]. Overall, 78 patients (91.8%) attained reperfusion. Successful reperfusion (mTICI ≥2b) was achieved in 93.0% of RESCUE-treated cases versus 90.5% of Solumbra-treated cases (*p* = 1.000). The FPR rate was markedly higher with RESCUE (37/43, 86.0%) than with Solumbra (26/42, 61.9%; *p* = 0.011). sICH occurred in one patient of the RESCUE group (2.3%) and two patients of the Solumbra group (4.8%). At 90 days, a favorable functional outcome (mRS ≤ 2) was seen in 26 RESCUE recipients (60.5%) and 24 Solumbra recipients (57.1%). The rate of excellent functional recovery (mRS ≤ 1) was numerically higher in the RESCUE group (37.21%, 16/43) compared with the Solumbra group (28.57%, 12/42) (see Figure 3). No significant between-group differences were detected in the number of thrombectomy passes, distal embolization, vessel dissection, perforation, 90-day mortality, or procedural complications (Table 2).

**Table 2:** Primary and secondary clinical outcomes of the study population.

Outcome	Total (*n* = 85)	RESCUE group (*n* = 43)	Solumbra group (*n* = 42)	*p* value
FPR, *n* (%)	63 (74.1)	37 (86.0)	26 (61.9)	0.011*
mTICI grade ≥2b recanalization, *n* (%)	78 (91.8)	40 (93.0)	38 (90.5)	0.373
90-day favorable outcome (mRS 0–2), *n* (%)	49 (57.6)	26 (60.5)	24 (57.1)	0.483
No. of thrombectomy passes, median (IQR)	1.00 (1.0, 2.0)	1.00 (1.0, 2.0)	1.00 (1.0, 2.0)	0.434
Distal embolization, *n* (%)	3 (3.5)	1 (2.3)	2 (4.8)	0.615
Vessel dissection, *n* (%)	6 (7.06)	3 (6.98)	3 (7.14)	1.000
Perforation, *n* (%)	0	0	0	—
Early reocclusion within 24 h, *n* (%)	4 (4.7)	2 (4.7)	2 (4.8)	1.000
Rescue angioplasty, *n* (%)	26 (30.1)	22 (58.1)	24 (57.1)	0.926
Rescue stenting, *n* (%)	9 (10.59)	5 (11.63)	4 (9.52)	1.000
sICH, *n* (%)	3 (3.5)	1 (2.3)	2 (4.8)	1.000
90-day mortality, *n* (%)	2 (2.4)	0 (0.0)	2 (4.8)	0.237

Data are presented as n (%) for categorical variables, and as median (IQR) for continuous variables.

mTICI, modified Thrombolysis in Cerebral Infarction; mRS, modified Rankin Scale; sICH, symptomatic intracranial hemorrhage.

*Indicates statistical significance (*p* < 0.05).

## Discussion

Findings from this study indicate that the RESCUE technique serves as a safe and effective endovascular option for individuals with refractory vessel blockages and suspected underlying ICAS-LVO. Application of the RESCUE approach in ICAS-LVO patients not only reduced the interval from arterial puncture to initial recanalization but also enhanced the FPR rate within the RESCUE cohort. Additionally, successful reperfusion (mTICI 2b-3) was attained in 93.0% of patients treated with the RESCUE technique compared with 90.5% of those receiving treatment with the Solumbra technique, a rate exceeding previously published figures (20).

The Solumbra technique, which integrates a stent retriever with thrombus aspiration, generally achieves satisfactory outcomes during MT (7). Nevertheless, individuals with ICAS-LVO frequently respond poorly to standard thrombectomy, leading to suboptimal recanalization success, elevated rates of reocclusion, and an increased likelihood of arterial dissection. Our previously proposed SPACEMAN (*S*tent-*P*ass-*A*spiration-res*C*u*E*-*M*icrowire-*A*ngioplasty) technique for ICAS-LVO also achieved favorable outcomes (17). Compared with SPACEMAN, the most distinctive feature of the RESCUE technique is the local injection of tirofiban through an aspiration catheter after stent positioning and deployment across the lesion. By delivering focal antiplatelet therapy under distal protection, this approach reduces the risk of distal embolization, improves reperfusion rates, and minimizes systemic adverse effects.

For acute non-cardioembolic ischemic stroke patients treated with intravenous thrombolysis but not undergoing endovascular intervention, early tirofiban administration has been demonstrated to enhance clinical outcomes without elevating the risk of serious adverse events (21). For patients with AIS-LVO undergoing endovascular thrombectomy, intravenous tirofiban infusion prior to the procedure did not significantly affect 90-day disability severity, suggesting no benefit from routine pre-thrombectomy intravenous tirofiban (9). Nevertheless, subgroup analyses indicated that tirofiban may confer benefit to patients with ICAS-LVO by increasing the rate of first-pass recanalization (22). Intra-arterial local administration of tirofiban may further improve recanalization efficacy and reduce complication rates (8, 12).

Although there was no statistically significant difference in dichotomized favorable 90-day functional outcomes between the two groups (60.5% vs. 57.1%, *p* = 0.483), ordinal mRS distribution analysis revealed a beneficial trend of functional recovery in the RESCUE group. The absence of statistical significance may be attributed to the limited sample size in this study. Among our study population, 24 patients (28.6%) underwent endovascular therapy following intravenous thrombolysis. Safety endpoint analyses revealed no statistically significant intergroup differences in the incidence of procedure-related complications and long-term adverse events. The median number of thrombectomy passes was identical between the two cohorts. For intraoperative vascular injuries, comparable rates of distal embolization (2.3% vs. 4.8%, *p* = 0.615) and vessel dissection (7.0% vs. 7.1%, *p* = 1.000) were observed in the RESCUE and Solumbra groups, and no vessel perforation occurred in any patient during the procedure. Moreover, the two arms exhibited similar rates of early reocclusion within 24 h, symptomatic intracranial hemorrhage (sICH), and all-cause mortality at 90 days.

Collectively, the RESCUE protocol did not raise the risks of intraoperative vascular trauma, distal thrombus migration, early vessel reocclusion, intracranial hemorrhage, or long-term mortality compared with conventional Solumbra thrombectomy. Despite its advantages of higher first-pass reperfusion (FPR) rates and shortened procedural duration, the RESCUE technique demonstrated a safety profile equivalent to standard mechanical thrombectomy for patients with ICAS-LVO. The overall incidences of sICH and death were 3 (3.6%) and 2 (2.4%), respectively. Previous investigations have likewise reported that, following unsuccessful thrombectomy in ICAS-LVO patients, intra-arterial and/or intravenous tirofiban can successfully preserve vascular recanalization and function as a rescue option before angioplasty (11, 13). The ANGEL-REBOOT trial remains the only worldwide multicenter randomized controlled trial specifically designed for endovascular treatment of ICAS-LVO (10). It recommended intraoperative and postoperative tirofiban administration whenever severe residual stenosis of the target vessel exists after thrombectomy, regardless of the degree of revascularization. When compared with tirofiban treatment, patients treated with rescue angioplasty face an elevated risk of symptomatic intracranial bleeding, parenchymal hematoma type 2 hemorrhage, and procedure-related arterial dissection.

The RESCUE BT trial demonstrated that preoperative intravenous tirofiban pretreatment yields limited overall clinical benefits and only shows potential efficacy in the ICAS-LVO subgroup, with inherent limitations in administration timing and delivery route (9). Differently, this study adopted an intra-arterial tirofiban rescue strategy after Retrievable stenting, which breaks through the research scope and technical bottlenecks of conventional rescue BT therapy via a distinct mechanism.

The RESCUE technique offers several key advantages. Initially, a stent retriever is positioned across the lesion to establish a bypass channel, while focal endovascular tirofiban is infused to augment local antiplatelet activity and facilitate softening of residual atherothrombotic material. Next, the aspiration catheter is navigated through the proximal thrombus along the stent retriever and advances into the distal vessel under continuous suction. Because clot extraction is performed first, this approach avoids the time that would otherwise be consumed managing reocclusion or arterial dissection during conventional stent retrieval. Third, a 300-cm microguidewire is advanced beyond the occlusion via the aspiration catheter; after the aspiration catheter is withdrawn to a position proximal to the blockage, angiography is performed. This sequence enables prompt implementation of rescue therapy after comprehensive lesion assessment. Rescue options include detachment of the stent retriever or balloon dilation in suitable candidates. The RESCUE technique is distinct from other combined aspiration-stent retriever approaches, including Solumbra, CAPTIVE (Continuous Aspiration Prior to Intracranial Vascular Embolectomy), SAVE (Stent-retriever Assisted Vacuum-locked Extraction), and ARTS (Aspiration-Retriever Technique for Stroke) (6, 15, 23, 24). It is also distinct from techniques involving intra-arterial tirofiban infusion after stent retriever deployment, such as SPACEMAN and SAIL (Stent retriever AssIsted Lysis) (13, 17).

This RESCUE technique requires multi-device intervention and individualized intra-arterial tirofiban administration, which heavily relies on operator proficiency and institutional resources and exhibits a clear learning curve. It integrates sophisticated intervention, personalized medication, and perioperative risk control, demanding solid expertise in cerebrovascular intervention. Inexperienced operators may incur procedural errors, impairing efficacy and increasing complication risks. Restricted by resource conditions, this technique is currently feasible mainly in well-established stroke centers but is limited in primary hospitals. Standardized protocols, professional training, and hierarchical technical support can shorten the learning curve and improve its generalizability across diverse medical centers.

Several limitations of the present work warrant acknowledgment. First, the sample size was adequate for the primary outcome but underpowered for secondary functional outcomes, leading to potential type II errors. Future studies will calculate targeted sample sizes for all endpoints and adopt larger cohorts to improve statistical efficacy. Second, patients with posterior circulation occlusion only accounted for 15.3%, and the insufficient sample size of this subgroup precluded statistical intergroup comparisons; therefore, quantitative analyses of therapeutic efficacy differences between anterior and posterior circulation subgroups were not performed. Third, the follow-up period was too short for patients receiving stents. The long-term advantages and potential risks of the RESCUE technique for treating ICAS-LVO require further investigation in future studies, and operator-dependent technical learning curve restricting broad external validity of the RESCUE technique.

## Conclusion

The RESCUE technique represents a new, safe, and effective endovascular treatment paradigm for AIS patients with ICAS-LVO, incorporating an aspiration catheter, a stent retriever, and a systematic rescue protocol. However, larger prospective investigations are needed to supply more robust evidence supporting its routine clinical use.

## Data Availability

The original contributions presented in the study are included in the article/supplementary material, further inquiries can be directed to the corresponding author.
